# Exome sequencing identifies a novel mutation in PIK3R1 as the cause of SHORT syndrome

**DOI:** 10.1186/1471-2350-15-51

**Published:** 2014-05-02

**Authors:** Clea Bárcena, Víctor Quesada, Annachiara De Sandre-Giovannoli, Diana A Puente, Joaquín Fernández-Toral, Sabine Sigaudy, Anwar Baban, Nicolas Lévy, Gloria Velasco, Carlos López-Otín

**Affiliations:** 1 Departamento de Bioquímica y Biología Molecular, Facultad de Medicina, Instituto Universitario de Oncología, Universidad de Oviedo, 33006 Oviedo, Spain; 2 Aix-Marseille Université, Inserm UMR_S 910, Faculté de Médecine de Marseille, 13385 Marseille cedex 05, France; 3 AP-HM, Département de Génétique Médicale, Hôpital d’Enfants de la Timone, 13385 Marseille cedex 05, France; 4 Departamento de Genética, Hospital Universitario Central de Asturias, 33006 Oviedo, Spain; 5 Cardiology and Cardiosurgical Department, Bambino Gesú Children’s Hospital, 00165 Rome, Italy

**Keywords:** Aging, Diabetes, Insulin, Kinase, Lipodystrophy, Progeria

## Abstract

**Background:**

SHORT syndrome is a rare autosomal dominant condition whose name is the acronym of short stature, hyperextensibility of joints, ocular depression, Rieger anomaly and teething delay (MIM 269880). Additionally, the patients usually present a low birth weight and height, lipodystrophy, delayed bone age, hernias, low body mass index and a progeroid appearance.

**Case presentation:**

In this study, we used whole-exome sequencing approaches in two patients with clinical features of SHORT syndrome. We report the finding of a novel mutation in *PIK3R1* (c.1929_1933delTGGCA; p.Asp643Aspfs*8), as well as a recurrent mutation c.1945C > T (p.Arg649Trp) in this gene.

**Conclusions:**

We found a novel frameshift mutation in *PIK3R1* (c.1929_1933delTGGCA; p.Asp643Aspfs*8) which consists of a deletion right before the site of substrate recognition. As a consequence, the protein lacks the position that interacts with the phosphotyrosine residue of the substrate, resulting in the development of SHORT syndrome.

## Background

Rare syndromes are disorders that, separately, affect a reduced number of individuals in the world. The scarcity of patients and resources makes it very difficult to establish the molecular cause of these conditions. Despite these drawbacks, the increasing knowledge in molecular biology as well as the development of next-generation sequencing methods has allowed the identification of the genetic defects that cause some of these rare syndromes, such as Néstor-Guillermo Progeria syndrome [1] and Kabuki syndrome [2].

SHORT syndrome is a rare autosomal dominant condition whose name is the acronym of short stature, hyperextensibility of joints, ocular depression, Rieger anomaly and teething delay (MIM 269880) [3]. Other typical features are low birth weight, lipodystrophy, delayed bone age, inguinal hernias, low body mass index and a marked progeroid appearance characterized by wrinkled skin, a triangular face with a small chin, low-set posteriorly rotated ears and thin alae nasi. All these clinical features go along with a usually normal intellect [4]. Recently, four groups have independently reported the finding of mutations in *PIK3R1* as the primary cause of SHORT syndrome [5-8]. In this study, we describe the use of whole-exome sequencing technology to identify a novel *PIK3R1* mutation, as well as a point mutation already reported in this gene, in two patients with SHORT syndrome.

## Case presentation

### Methods

#### Exome sequencing

Written, informed consent was obtained from all subjects or from their legal representatives, before enrollment in the study. Both families (patient 1 and father of patient 2) also provided a written and informed consent for the publication of the images included in this article. The study protocol was approved by the ethics committee of the Hospital Universitario Central de Asturias, in compliance with the Helsinki Declaration. By the time this sequencing analysis was carried out, the genetic cause of SHORT syndrome was still unknown, what led us to perform an exome sequencing analysis. For this purpose, genomic DNA was extracted from peripheral blood leukocytes with a Qiagen kit according to the manufacturer’s instructions (QIAGEN, Germany). Exome capture was performed using a SureSelectXT Human All Exon 50 Mb Kit (Agilent). Briefly, 3 μg of genomic DNA were sheared with a Covaris S2 instrument and used for the construction of a paired-end sequencing library as described in the paired-end sequencing sample preparation protocol provided by Illumina. Enrichment of exonic sequences was then performed using the Sure Select Human All Exon 50 Mb Kit (Agilent Technologies) following the manufacturer’s instructions. Exon-enriched DNA was pulled down using magnetic beads coated with streptavidin (Invitrogen), followed by washing, elution and 18 additional cycles of amplification of the captured library. The enriched library was sequenced (2×76 bp) using a HiSeq 2000 instrument (Illumina).

#### Exome sequence data analysis

Sequence data were analyzed using a custom pipeline based on the *Sidrón* algorithm [9,10]. Reads were mapped to the human reference genome (GRCh37) using BWA with the sampe option, and a BAM file was generated for each sample using SAMtools. Optical or PCR duplicates were removed using the rmdup option of SAMtools. A first loose filter was used to eliminate any genomic position where variants were extremely unlikely. Then, each candidate variant was given an *S* score with *Sidrón*. If data from relatives were also available, they were incorporated at this step. Cutoff points were set depending on coverage (*cov*) as follows: positions with an S value lower than (−0.2583*cov + 2.6546) were considered homozygous. Positions with a coverage lower than 20 were considered heterozygous if their *S* value was higher than 5.807. Positions with a coverage higher than or equal to 20 were considered heterozygous if their *S* value was higher than (0.7019*cov - 9.6348). The rules to call a variant were: a) If a position is called as heterozygous, it is considered a heterozygous variant; b) If the most frequently read base is not the reference base and the position cannot be called as heterozygous, it is considered a variant; c) If the most frequently read base is not the reference base and the position is classified as homozygous, it is considered a homozygous variant. Variants present in dbSNP137 with a minor allele frequency higher that 0.01, or present in more than 2% of individuals of Spanish origin without previous history of progeroid syndrome for which exome data was available as part of the CLL-ICGC project, were discarded as common polymorphisms. Variants potentially affecting protein function, including non-synonymous variants, frameshifts in the coding sequence, or variants potentially affecting splicing, were identified with *Mutandis*, from the *Sidrón* pipeline [9,10].

#### Sanger sequencing

The mutations detected in the whole-exome analysis were validated through Sanger sequencing. A fragment of 307 bp from exon 19 of *PIK3R1* was PCR-amplified (5′-ATGGCTCCTGCACTCTTC AT-3′ and 5′-AAATCTTTGCCCCCAAAACT-3′) and then, Sanger sequencing was performed using the same primers on an ABI PRISM 3130×l Genetic Analyzer. In patient 1, sequence traces were analyzed with Mutation Surveyor (v.3.24, SoftGenetics).

### Results

#### Clinical report of two patients with SHORT syndrome

**Figure 1:**
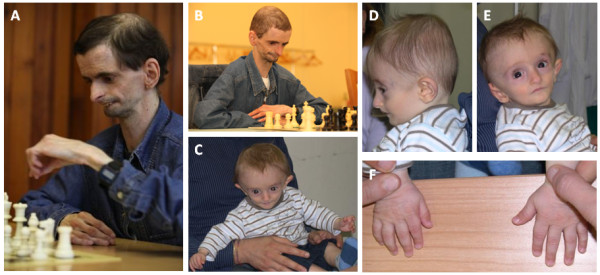
**Facial appearance of patient 1 (A and B) and patient 2 (C-F).** The appearance of the patients included in this study shows a clear premature aging phenotype, detectable from the first months of life (patient 2) and striking in adulthood (patient 1).

We studied 2 independent families from distant origins with one affected member in each family (Table 1). Both families gave their informed consent prior to their inclusion in the study. The first patient studied presented characteristics of SHORT syndrome at an early age, and has already developed all the symptoms associated with this syndrome so far. He was the third child from a Spanish couple, both parents and both siblings being healthy and with no relevant clinical family history. At conception, his father was 48-year-old and his mother 39-year-old. He was born at terminus but with intrauterine growth retardation (1,600 g at birth). The patient was diagnosed with an atypical progeroid syndrome at the age of 2 years and 6 months, presenting at that moment with low height (82 cm, <3^rd^ percentile), low weight (7,700 g, <3^rd^ percentile), a small head circumference (46 cm, <3^rd^ percentile), lipodystrophy and wrinkled skin (especially on the hands). At 13 years of age, he was diagnosed with diabetes and axillary acanthosis nigricans. His facies shows a pronounced progeroid phenotype with triangular face, ocular depression, a small chin and thin alae nasi (Figure 1A-B). Patient 1 is nowadays 31-year-old and he is short (153.7 cm, <3^rd^ percentile), very thin (28 kg, <3^rd^ percentile) and has hypercholesterolemia (LDL: 181 mg/dL). He has got an extremely weak constitution and a high pitched voice. He has also developed Rieger anomaly with Axenfeld syndrome, as well as glaucoma and severe myopia. As typical in SHORT syndrome, this patient also has hyperextensibility of joints with hip and knee osteoarthritis. He presents normal renal and hepatic function, but he has pulmonary hypoplasia and pulmonary valve stenosis that has never required surgical intervention. His intellectual and psychomotor development has always been in normal range.

**Table 1:** Clinical features of the two analyzed patients with SHORT syndrome

	**Patient 1**	**Patient 2**
**General characteristics**		
Gender	Male	Male
Father age at conception	48-year-old	33-year-old
Weight at birth	1 600 g	2 030 g
Height at birth	Not reported	44.5 cm
Age at assessment	2 years 6 months	6 ½ months
Height at assessment	82 cm (<3^rd^ percentile)	62 cm (<3^rd^ percentile)
Weight at assessment	7 700 g (<3^rd^ percentile)	4 860 g (<3^rd^ percentile)
Head circumference at assessment	46 cm (<3^rd^ percentile)	41.5 cm (4.5^th^ percentile)
**Facies**		
Progeroid appearance	yes	yes
Triangular face	yes	yes
Ocular depression	yes	yes
Small chin	yes	yes
Low-set posteriorly rotated ears	no	yes
Thin alae nasi	yes	yes
**Other SHORT syndrome characteristics**		
Intrauterine growth retardation	yes	yes
Rieger anomaly	yes (with Axenfeld syndrome). Glaucoma and severe myopia	no
Lipodystrophy	yes	yes
Wrinkled skin	yes (on the hands)	yes (on the hands)
Hyperextensibility of joints	yes (artrosis evolution: hip and knee)	no
Inguinal hernia	no	no
Insulin resistance	yes, with axillary acanthosis nigricans	N/A
Bone age	normal (osteoporosis evolution)	Normal
Psychomotor development	Normal	Normal
**Other findings**		
	Pulmonary valve stenosis, pulmonary hypoplasia, hypercholesterolemia	Gastro-esophageal reflux, patent anterior fontanelle, patent foramen ovale
***PIK3R1 *** **mutation found**	c.1929_1933delTGGCA	c.1945C > T

Patient 2 is an Italian child with a mild phenotype resembling SHORT syndrome. He was born on the 38^th^ week of pregnancy by urgent cesarean section. At conception, his father was 33-year-old and his mother 34-year-old. At birth, he weighed 2,030 g (<3^rd^ percentile), measured 44.5 cm (3^rd^ percentile) and his occipitofrontal circumference was 31 cm (3^rd^ percentile). However, he obtained a 9–9 score in the APGAR test. During pregnancy, all screening tests were within the normal range (fetal movement and prenatal screening tests for Down syndrome), but from week 25^th^ of gestation growth retardation with abnormal uterine doppler was noted. Due to intrauterine growth retardation, he was subjected to ultrasound screening for 30 months after birth, with normal results in all cases. He had a poor sucking reflex for the first two months after birth, which improved later. At 6 months of age, he was diagnosed with an atypical progeroid syndrome, evocative of Wiedemann-Rautenstrauch syndrome (MIM 264090), presenting by that time low parameters for height (62 cm, <3^rd^ percentile), weight (4,860 g, <3^rd^ percentile) and head circumference (41.5 cm, 4.5^th^ percentile). Patient 2 was evaluated again at 6 months of age and showed lipodystrophy with wrinkled skin on the hands (Figure 1C-F). He also had a triangular face, ocular depression, a small chin and thin alae nasi. Additionally, as other patients affected with SHORT syndrome, patient 2 showed low-set posteriorly rotated ears. Ophthalmologic examination of this patient was normal and he did not show joint hyperextensibility. He suffered from gastro-esophageal reflux that was treated conservatively. He also had patent anterior fontanelle and patent foramen ovale. Patient 2 also had a normal intellectual and psychomotor development.

### Exome sequencing reveals different PIK3R1 mutations in SHORT syndrome patients 1 and 2

Whole-exome sequencing was performed on patient 1 as well as on his mother and sister. DNA from patient’s father was not available for study. Overall, more than 89% of the exome regions were considered callable. We first confirmed that no variants affecting genes previously associated with progeroid syndromes were present in the exome of the patient. After removing common polymorphisms and variants present in mother and sister with the same zygosity, we identified 126 variants potentially affecting protein function due to the introduction of missense, nonsense, splicing or frameshifts in the coding regions, and not present in dbSNP137. In parallel studies, we obtained the exome sequence of patient 2, with more than 85% of the bases classified as callable. We identified 58,122 variants, out of which 7,711 were predicted to affect the sequence of a protein. Furthermore, only 166 of those variants were absent in dbSNP137 or in a local database containing constitutive variants from Spanish individuals. After comparing the sets of proteins affected by the candidate variants from both individuals, we found that only *PIK3R1* and *ZNF276* were selected in both patients. Moreover, both *ZNF276* variants affect only a minor transcript with no CCDS identifier (ENST00000446326). Therefore, this analysis singled out *PIK3R1* as the most likely candidate driver of the disease.

**Figure 2:**
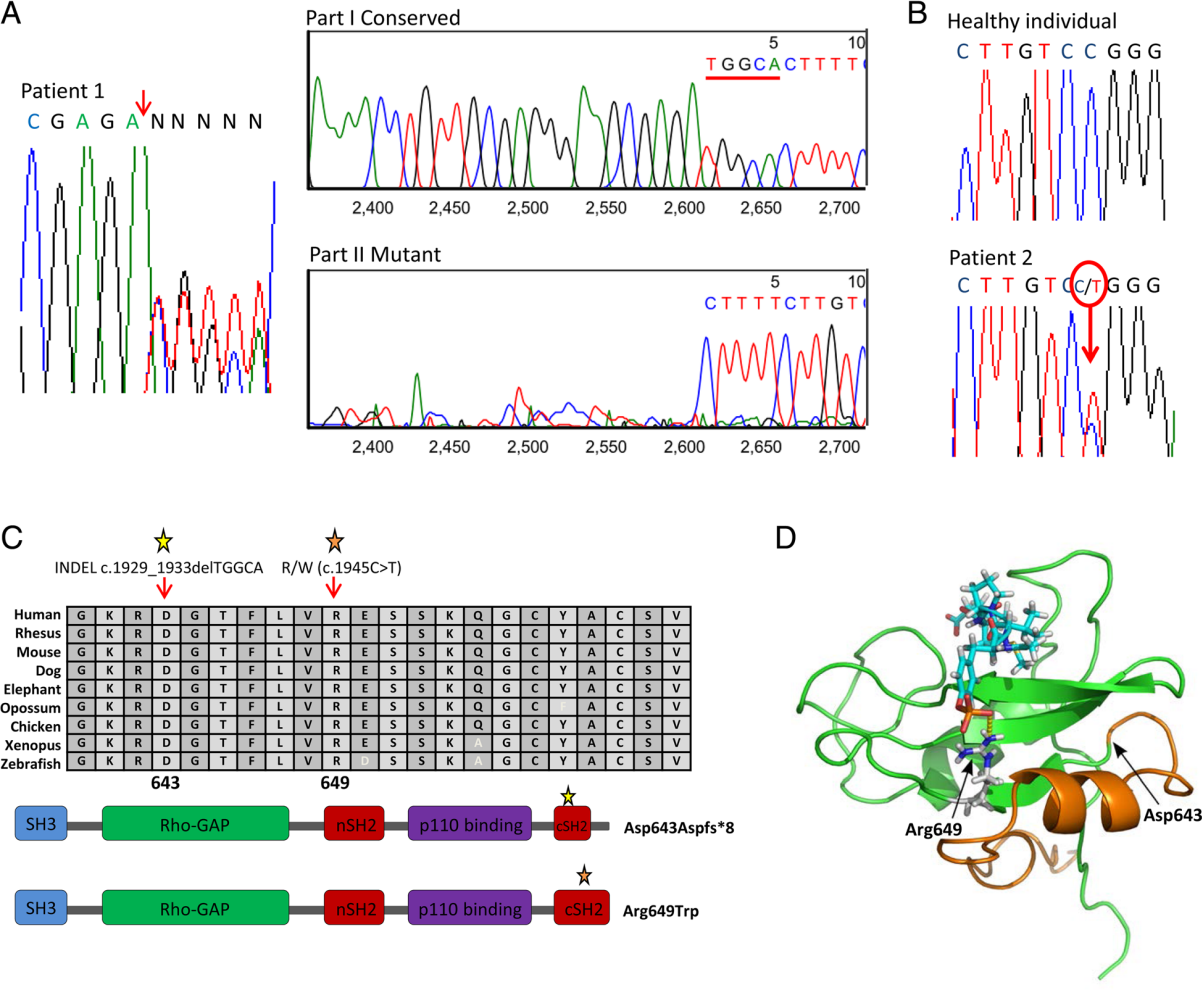
**Sanger sequencing of *PIK3R1 *(A and B), schematic representation of p85α (C) and spatial modeling of the mutated protein (D).** Electropherograms from patients 1 **(A)** and 2 **(B)** showing the corresponding mutations in *PIK3R1*. In patient 1, deletion of 5 nucleotides was detected with Mutation Surveyor software. **(C)** Schematic representation of the protein mutated in SHORT syndrome patients. The two mutations described in this article are outlined by a red and a yellow star. Both mutations are located in an SH2 region. In patient 1, the mutation results in a truncated protein, whereas in patient 2, it causes an amino acid residue change. **(D)** Spatial localization of the mutated residues. The mutated residues are shown on the SH2 domain contained in the PDB structure 1PIC. The truncated form resulting after mutation p.Asp643Aspfs*8 is shown in orange. Arginine 649 is shown interacting (yellow dashed line) with the phosphate group of Acetyl-pTyr-Val-Pro-Met-Leu (blue and red sticks). The picture was rendered with PyMOL (v.0.99rc6).

The variant affecting *PIK3R1* in patient 1 is a heterozygous deletion of 5 nucleotides (c.1929_1933delTGGCA) that was confirmed by Sanger sequencing and analysis with Mutation Surveyor (Figure 2A). This indel causes the disruption of the reading frame from position 643 of the protein and the generation of an in-frame premature stop codon seven residues downstream (p.Asp643Aspfs*8) (Figure 2C). This deletion was absent in both mother and sister of patient 1. In patient 2, we found the variant c.1945C > T, which causes the change of arginine to tryptophan in amino acid 649 of PIK3R1 protein (p.Arg649Trp). This mutation was also confirmed by Sanger sequencing (Figure 2B) and was absent in the patient’s parents. We also performed a structural model of the protein showing the two mutations described in this study (Figure 2D). The p.Arg649Trp (c.1945C > T) mutation affects a position that is involved in the interaction with the phosphotyrosine residue of the substrate, as shown by the proximity of this residue to the functional group of a substrate-mimic molecule. The p.Asp643Aspfs*8 (c.1929_1933delTGGCA) mutation of patient 1 truncates the protein right after the first alpha helix and before this site of substrate recognition. As a result, this alpha helix might fold properly but the resulting protein lacks the position that interacts with the phosphotyrosine residue of the substrate.

### Discussion

We describe herein the whole-exome sequence analysis of two patients, one Spanish and one Italian, presenting with a progeroid phenotype whose clinical features fit a SHORT syndrome diagnosis [4]. Both patients share intrauterine growth retardation, short stature, low body mass index, microcephaly, triangular face and wrinkled skin, and the older one also presents insulin resistance, Rieger anomaly and hyperextensibility of joints. We show evidence of the causative role of mutations in *PIK3R1* in SHORT syndrome, adding to the recently published papers that show mutations in this gene in other patients with this progeroid syndrome [5-8].

The first patient described herein presents a novel frameshift mutation in *PIK3R1* that has no similarity with any mutation reported before in the context of SHORT syndrome. It consists of a heterozygous deletion of 5 nucleotides from coding position 1929 to 1933 of the *PIK3R1* gene. The deletion of these 5 nucleotides (c.1929_1933delTGGCA) elicits the disruption of the reading frame and the appearance of a stop codon 7 residues downstream, leading to a truncated protein. The absence of this deletion in his mother and sister and the lack of any SHORT syndrome phenotype in his father strongly suggest the occurrence of a *de novo* mutation in this patient. The second patient presents a mutation that is recurrent in other individuals with SHORT syndrome [5-8], which consists of a *de novo* heterozygous C > T transition at coding position 1945 of *PIK3R1*. This transition results in a change from arginine to tryptophan at position 649 of the mature protein.

*PIK3R1* encodes the subunit p85α of the PI3K kinase. This protein kinase is part of an important metabolic pathway that induces cell growth, proliferation, protein synthesis and apoptosis restraint, among other effects [11]. As it has been recently published, *PIK3R1* mutations seem to induce the downregulation of the Akt/mTor pathway [5-7], which could explain the small stature and weight of these patients as well as the insulin resistance that they usually suffer from.

## Conclusion

In summary, by using whole-exome capture and next-generation sequencing, we report *PIK3R1* as the gene implicated in SHORT syndrome in two patients, further supporting the recently published works about this syndrome [5-8]. Additionally, we report a novel mutation related to this syndrome in a Spanish patient. Furthermore, the fact that *PIK3R1* encodes a protein with an important role in the regulation of a metabolic pathway may establish a new group of accelerated aging disorders, which until now were mainly caused by alterations in the nuclear envelope or by defects in the DNA repair systems [12-14]. This discovery may also yield new insights into the mechanisms of human normal aging [15], especially in relation to metabolic alterations occurring during this process.

## Competing interests

The authors declare that they have no conflict of interest.

## Authors’ contributions

CB, VQ, GV performed and analyzed the exome-sequencing and validated the mutations obtained. AdS-G performed parent’s analysis of one patient. JF-T and AB contacted patients and collected the samples. AdS-G, SS, NL and JF-T studied and diagnosed the patients. DAP collaborated in the preparation of the samples. CL-O and GV conceived and supervised the work. CB and CL-O designed and wrote the paper. All authors read and approved the final manuscript.

## Pre-publication history

The pre-publication history for this paper can be accessed here:

http://www.biomedcentral.com/1471-2350/15/51/prepub
